# Oral Health Status, Salivary Characteristics, and Cariogenic Bacterial Levels in Children and Adolescents with Haemophilia

**DOI:** 10.3390/jcm15218385

**Published:** 2026-10-28

**Authors:** Kubra Yilmaz Calisir, Buse Nur Gok, Dilek Aksu Guler, Funda Tayfun Kupesiz, Osman Alphan Kupesiz, Huseyin Karayilmaz

**Affiliations:** 1 Private Practice, 07070 Antalya, Turkey; dtkubiss@outlook.com; 2 Department of Paedodontics, Faculty of Dentistry, Akdeniz University, 07058 Antalya, Turkey; buseeegok@gmail.com; 3 Private Practice, 07160 Antalya, Turkey; dilekaksuguler@gmail.com; 4 Division of Hematology, Department of Paediatrics, Faculty of Medicine, Akdeniz University, 07070 Antalya, Turkey; fundatayfunkupesiz@akdeniz.edu.tr (F.T.K.); akupesiz@akdeniz.edu.tr (O.A.K.)

**Keywords:** Haemophilia, oral health, dental caries, saliva, *Streptococcus mutans*, Pediatric dentistry

## Abstract

**Objective:** To evaluate oral health status, salivary characteristics, and cariogenic bacterial levels in children and adolescents with haemophilia, and to compare these parameters with those of healthy peers. **Methodology:** The study included 43 children and adolescents with haemophilia and 40 healthy controls. Both groups were assessed at baseline, and 34 participants with haemophilia were reassessed after six months; controls were assessed only at baseline. Stimulated saliva samples were analysed for flow rate, buffering capacity, and levels of *Streptococcus mutans* and *lactobacilli*. **Results:** At baseline, the haemophilia group exhibited significantly higher OHI, PI, GI, calculus index, and DMFT scores (*p* < 0.05). At six months, OHI, PI, and calculus index decreased and no longer differed significantly from the controls’ baseline values, whereas GI and DMFT remained elevated. *S. mutans* levels were significantly higher at baseline but decreased significantly over time, and *lactobacilli* levels also decreased significantly (*p* = 0.001) and were lower than the controls’ baseline values at six months. **Conclusions:** Children with haemophilia demonstrate compromised oral health; oral hygiene parameters improved after preventive follow-up, although, without a longitudinal control group, these changes cannot be attributed to the intervention alone. These findings underscore the importance of early dental care in this high-risk population.

## 1. Introduction

Haemophilia A and B are inherited X-linked bleeding disorders caused by deficiencies of factor VIII (FVIII) and factor IX (FIX), respectively [1]. Haemophilia affects approximately 400,000 individuals worldwide [2]. In dental practice, it requires special attention because intraoral bleeding may be severe or even the first diagnostic sign, particularly in mild cases [3].

The main oral health problems in individuals with haemophilia are dental caries and periodontal disease. Fear of bleeding may discourage oral hygiene practices and increase the risk of caries, gingivitis, and periodontitis [4]. Because oral tissues are highly vascular and fibrinolytic, these patients are prone to intraoral bleeding [5]. Bleeding occurs most commonly in the labial frenulum, tongue, and buccal mucosa, and is more frequent in severe haemophilia [3,6]. Spontaneous bleeding may occur because of dilated capillaries in thin gingival tissues during tooth brushing, food impaction, or periodontal disease [4]. Fear of bleeding may lead to ineffective toothbrushing, exacerbating gingival inflammation and bleeding [6].

Patients with haemophilia therefore require specific precautions during oral examination and dental treatment. However, evidence on oral health in this population remains limited and inconsistent. Since oral diseases may worsen haemophilia-related complications, further well-designed studies are needed to improve clinical outcomes.

Data on the combined clinical, salivary and microbiological status of children with haemophilia, and on how these parameters change during a short follow-up after a preventive programme, are limited. Therefore, this study aimed to evaluate dental caries, oral hygiene status, salivary buffering capacity, salivary flow rate, and levels of *S. mutans* and *lactobacilli* in children and adolescents with haemophilia, and to compare these findings with healthy controls of similar age and sex. The study also aimed to support oral hygiene education and improvement strategies in this population.

## 2. Methodology

This study was approved by the Clinical Research Ethics Committee of Akdeniz University Faculty of Medicine (decision no. 845, 5 December 2018). Written and verbal informed consent was obtained from all participants and their parents.

### 2.1. Establishment of the Study Groups

This was a prospective, single-centre observational study, completed in 2020 as a specialty thesis. Children and adolescents with haemophilia and healthy controls were examined at baseline, and the participants with haemophilia were re-examined after six months. The study was approved by the Clinical Research Ethics Committee of Akdeniz University Faculty of Medicine on 5 December 2018 (decision no. 845). Participants were enrolled between March 2019 and March 2020, and the six-month follow-up examinations were completed by September 2020.

The study group included 4–17-year-old patients with haemophilia followed at the Department of Paediatrics, Division of Haematology, Akdeniz University Faculty of Medicine. The control group comprised systemically healthy children attending Akdeniz University Faculty of Dentistry for routine dental care. Children with systemic diseases other than haemophilia, or who had used systemic antibiotics, antidepressants, psychiatric drugs, or similar medications within the previous 3 months, were excluded. Participants were selected from the participants registered in the records of the Division of Haematology, Department of Paediatrics, who met the inclusion criteria and agreed to participate. The sample size was calculated with G*Power 3.1.9.4 (Heinrich Heine University, Düsseldorf, Germany) on the basis of the study by Evangelista et al. [1]. With an effect size of 1.92885, a significance level of 0.05, and a power of 0.95, a minimum of 26 participants per group was required. To allow for loss to follow-up and to increase statistical power, 43 participants with haemophilia and 40 healthy controls were included. Controls were recruited during the same period, taking the age and sex of the haemophilia group into account.

The haemophilia group consisted of 43 participants, including 33 with haemophilia A and 10 with haemophilia B. Among patients with haemophilia A, 27 had severe, 5 had moderate, and 1 had mild disease; among those with haemophilia B, 4 had severe, 4 had moderate, and 2 had mild disease. The control group included 40 healthy children.

### 2.2. Clinical Examinations and Intra-Rater Reliability

All examinations were performed by a single calibrated researcher. Before the study, clinical and microbiological measurements were repeated by the same researcher, a few days apart, in 14 boys aged 5–15 years who were randomly selected among the patients attending the Department of Paedodontics for oral examination and treatment. A single overall intra-class correlation coefficient (ICC) calculated from the pooled measurements was 0.913; outcome-specific coefficients, the ICC model and its confidence interval are not available. The clinical examiner and the person who read the bacterial tests were unaware of the participants’ group status.

Medical histories were recorded and clinical oral examinations were performed for all participants. Caries experience was assessed using the DMFT index for permanent teeth (sum of decayed, missing due to caries, and filled teeth) and the dft index for primary teeth (sum of decayed and filled teeth). DMFT was calculated for participants with at least one permanent tooth and dft for those with at least one primary tooth; children with mixed dentition were included in both indices. Unerupted, supernumerary, retained, and physiologically exfoliated teeth, and teeth missing for reasons other than caries, were not scored [7]. Dental caries was diagnosed according to the ICDAS II criteria; teeth with ICDAS codes 2–6 (from a distinct visual change in enamel to extensive cavitation) were counted as decayed in both the DMFT and the dft.

Oral hygiene was assessed using the simplified Oral Hygiene Index (OHI) of Greene and Vermillion [8] on teeth 16, 26, 11, 31, 36 and 46 (facial surfaces of teeth 16, 26, 11 and 31, and lingual surfaces of teeth 36 and 46). Plaque (debris, PI) and calculus were each scored from 0 to 3 according to the proportion of the tooth surface covered, and the OHI was obtained by adding the mean plaque and the mean calculus scores. When an index permanent molar had not erupted, the adjacent second primary molar was scored instead; when an index incisor (11 or 31) had not erupted, it was not scored. Participant-level scores were calculated as the mean of the scores of the teeth examined. Gingival inflammation was assessed using the Silness and Löe Gingival Index (GI) by probing the gingival sulcus [9].

### 2.3. Evaluation of Saliva Parameters

Saliva samples were collected between 9:00 and 11:00 a.m., at least 2 h after breakfast and after mouth rinsing with water. Stimulated saliva was collected after participants chewed paraffin gum for 2 min and then expectorated into a sterile container for 5 min. Salivary flow rate was recorded as mL/min. Salivary buffering capacity was assessed using the CRT Buffer kit (CRT Buffer Refill, Ivoclar Vivadent, Schaan, Liechtenstein). One millilitre of stimulated saliva was applied to the test strip and scored after 5 min by colour change. Salivary *S. mutans* and *lactobacilli* levels were determined using CRT^®^ Bacteria test kits (Ivoclar Vivadent AG, Schaan, Liechtenstein). Samples were spread onto the test media and incubated at 37 °C for 48 h (EN 300, Nüve, Ankara, Turkey). Bacterial density was categorised according to the manufacturer’s reference chart as absent (no bacterial growth), low (<10^5^ CFU/mL), moderate (10^5^ CFU/mL), or high (>10^5^ CFU/mL); these categories are semi-quantitative and do not represent exact counts [10].

### 2.4. Study Protocol

Both groups were examined at baseline. The control group was examined only once, as a cross-sectional reference, during the same period in which the participants with haemophilia were recruited. After the baseline examination, the controls received preventive advice and fluoride application, and no other dental treatment was provided to them within the scope of the study. As they therefore no longer represented an untreated reference group, they were not followed up. Participants with haemophilia were reassessed after six months, and in this group all clinical indices, DMFT/dft values, salivary flow rate, buffering capacity, and salivary *S. mutans* and *lactobacilli* levels were assessed at baseline and at six months.

After baseline evaluation, participants and parents received individual verbal oral hygiene education from a single specialist paediatric dentist at the end of the baseline examination, including a demonstration of the roll toothbrushing technique and dietary recommendations; it was given once and not repeated during follow-up. A children’s fluoride toothpaste was recommended (1000 ppm fluoride for children younger than 6 years, 1450 ppm for those aged 6 years and older). In addition, 5% NaF/xylitol varnish (ProShield, President Dental, Allershausen, Germany) was applied once at baseline, using the same protocol in all participants (cotton-roll isolation, 5-min contact time). Apart from these preventive measures, no other dental treatment (including professional cleaning, scaling, restorations, or extractions) was provided by the research team during the six-month follow-up. The short follow-up period was chosen to avoid prolonged postponement of the dental treatment needs of children with haemophilia.

Baseline and six-month values of the haemophilia group were compared with the control group’s single baseline measurement. The study therefore comprised a cross-sectional baseline comparison between children with haemophilia and healthy controls, followed by an uncontrolled before-and-after assessment within the haemophilia group. Participants who received dental treatment during the follow-up period were excluded from the follow-up analysis.

### 2.5. Statistical Analysis

The data were analysed using SPSS version 18.0 for Windows (Chicago, IL, USA). Descriptive statistics, ICC reliability analysis, and comparative tests were performed. In cases where parametric assumptions were met, as assessed by Levene’s test for homogeneity of variances and the Shapiro–Wilk test for normality, the Student’s *t*-test was used for quantitative data. Baseline and six-month measurements in the haemophilia group were treated as paired observations and compared with the paired-samples *t*-test (quantitative data) or the Wilcoxon signed-rank test (ordinal categories). Ordinal categories (salivary buffering capacity, *S. mutans*, and *lactobacilli* levels) were compared between the haemophilia group and the controls with the Kruskal–Wallis test. For subgroup comparisons according to haemophilia severity, one-way ANOVA and Tukey’s HSD test were applied for quantitative data and the Kruskal–Wallis test for ordinal categories; these analyses were exploratory and were not adjusted for multiplicity. Longitudinal analyses were restricted to the 34 participants with paired observations. The tests used are also stated in the table titles. Results were evaluated at a 95% confidence level, and *p* < 0.05 was considered statistically significant. No adjustment for multiple comparisons was made, and all *p* values are nominal; results close to the 0.05 threshold should be regarded as borderline and interpreted with caution.

## 3. Results

The study included 43 male participants with haemophilia aged 4–17 years (mean age: 9.90 ± 4.38 years) and 40 healthy male controls (mean age: 9.62 ± 2.34 years), with no significant age difference between groups (*p* = 0.719). Preventive oral health behaviour in the haemophilia group was limited: 51.2% brushed only occasionally and 14.0% never brushed, while 58.1% visited the dentist only when in pain and 30.2% only occasionally.

### 3.1. Baseline Comparisons

At baseline, OHI, PI, GI, calculus index, and DMFT were significantly higher in the haemophilia group than in controls (*p* < 0.05), whereas dft was higher but not significant (*p* = 0.715). Salivary flow rate did not differ significantly between groups (*p* = 0.869) (Table 1). No significant differences were found in salivary buffering capacity or *lactobacilli* levels (*p* = 0.398 and *p* = 0.204, respectively), while *S. mutans* levels were significantly higher in the haemophilia group (*p* = 0.047) (Table 2).

### 3.2. Six-Month Comparisons with Controls

Of the 43 participants with haemophilia, 34 completed the six-month follow-up. Nine participants (20.9%) did not complete the follow-up: four did not wish to attend the hospital because of the COVID-19 pandemic, one had moved to another city, one reported being unable to continue for socioeconomic reasons, and three started orthodontic treatment and therefore received dental treatment, so they were excluded from the follow-up analysis. Among followed participants, 25 (73.5%) had haemophilia A and 9 (26.5%) had haemophilia B.

At six months, no statistically significant differences from the controls’ baseline values were detected in OHI, PI, calculus index, dft, salivary flow rate, salivary buffering capacity, or *S. mutans* levels (*p* > 0.05) (Table 1 and Table 2). *Lactobacilli* levels, however, were significantly lower in the haemophilia group than in controls (*p* < 0.001), with 52.9% of the haemophilia group in the absent category and none in the high category (Table 2). GI and DMFT remained significantly higher in the haemophilia group (*p* = 0.0001 and *p* = 0.007, respectively), while the baseline difference in *S. mutans* levels was no longer present.

### 3.3. Changes from Baseline to Six Months in the Haemophilia Group

In the haemophilia group, OHI, PI, and GI values decreased significantly from baseline to six months (*p* = 0.0001 for each), whereas the decrease in the calculus index (*p* = 0.022) was borderline. Salivary flow rate and the *S. mutans* and lactobacilli levels also changed significantly (*p* = 0.002, *p* = 0.012, and *p* = 0.001, respectively), while the change in buffering capacity (*p* = 0.046) was borderline. No statistically significant change was detected in DMFT and dft (*p* = 0.161 and *p* = 0.087, respectively) (Table 3 and Table 4).

### 3.4. Analysis According to Disease Severity

When stratified by disease severity as severe, moderate, and mild haemophilia, the severity subgroups showed no significant baseline differences in OHI, PI, GI, calculus index, DMFT, dft, salivary flow rate, salivary buffering capacity, *S. mutans*, or *lactobacilli* levels (*p* > 0.05). These analyses were exploratory because of the small subgroups (31, 9 and 3 participants at baseline; 24, 8 and 2 at six months). At six months, salivary flow rate differed among the subgroups (overall *p* = 0.015), with mean values of 0.67 ± 0.24 mL/min in the severe (*n* = 24), 0.95 ± 0.09 mL/min in the moderate (*n* = 8) and 0.55 ± 0.21 mL/min in the mild group (*n* = 2); this finding was not adjusted for multiplicity (Table 5 and Table 6).

## 4. Discussion

This study showed that children with haemophilia had poorer oral hygiene, higher gingival inflammation, greater caries experience, and higher *S. mutans* levels than healthy controls at baseline. In the haemophilia group, oral hygiene parameters improved significantly during the six-month follow-up, although this follow-up had no control group.

Children with chronic conditions may be more susceptible to dental disease due to disrupted oral hygiene, limited dental care, and caregiver priorities [11]. In haemophilia, intraoral bleeding may prompt dental attendance or diagnosis; severe intraoral bleeding has been reported as the diagnostic trigger in 14% of all cases and up to 30% of mild cases [3]. Therefore, early oral health assessment and preventive strategies are essential [11,12].

Given the X-linked recessive inheritance of haemophilia, most affected individuals are male [1]. Accordingly, the present cohort consisted exclusively of male participants, consistent with previous reports [13,14,15,16]. The age range (4–17 years) was also consistent with paediatric haemophilia studies [1,17,18]. The predominance of haemophilia A over haemophilia B reflected the distribution reported in the literature [12,13,18].

In individuals with haemophilia, periodontal disease may increase gingival bleeding because fragile, superficially located capillaries can bleed after even minor mechanical stimuli. Fear of bleeding may therefore impair toothbrushing, promoting plaque accumulation and gingival inflammation [6]. The literature reports conflicting findings on oral hygiene in patients with haemophilia, with studies showing better oral hygiene [11,19], poorer outcomes [15,16,18,20], or no significant differences compared with healthy controls [21]. These discrepancies may reflect methodological, population, behavioural, and socioeconomic differences. In the present study, baseline PI, OHI, and calculus index values were significantly higher in the haemophilia group compared with controls, supporting findings that indicate poorer oral hygiene in this population [15,16,18,20,22]. After oral hygiene instruction and fluoride varnish application, these values improved significantly at six months, and the differences from the control group’s baseline values were no longer statistically significant. Although longitudinal data in haemophilia populations remain limited [17], these findings suggest that targeted education and motivation may be associated with short-term improvements; however, without a control group followed over time and with possible co-interventions, the changes cannot be attributed to the preventive programme alone.

Coagulation disorders may further predispose individuals with plaque-induced inflammation to periodontal breakdown and alveolar bone loss [19]. GI, in which bleeding is a key indicator, is widely used to assess gingival inflammation [9]. Previous studies on GI in participants with haemophilia have reported heterogeneous findings, including higher values [18,21,22] or similar/lower values compared with healthy individuals [11]. In the present study, GI scores were significantly higher in the haemophilia group, consistent with reports of increased gingival inflammation in these participants [18,21,22]. Although GI scores decreased at six months, they remained higher than in controls. Because bleeding contributes to the GI, the higher values in the haemophilia group may reflect plaque-related inflammation, haemostatic differences, or both, rather than delayed gingival recovery alone. Longitudinal data on GI in patients with haemophilia are limited; Gaddam et al. [17] reported significant reductions at 3 months but no further improvement between 3 and 6 months, partially supporting our findings.

Dental caries remains common in childhood, and DMFT/dft indices help evaluate caries risk in patients with haemophilia [21]. Caries prevalence and DMFT/dft values in patients with haemophilia vary considerably across studies [16,21]. Some report lower caries prevalence [11,12,16,23], whereas others report higher prevalence or no significant differences compared with controls [13,20,21,22]. These inconsistencies may reflect differences in preventive care, dental visits, oral hygiene, diet, and socioeconomic status [1,11,12,16,20,21,23]. In the present study, baseline DMFT values were significantly higher in the haemophilia group, whereas dft values were higher but not statistically significant. These findings agree with studies reporting increased DMFT values in patients with haemophilia [14,20,21,22], and no significant difference in dft values [11,15,18,22]. This pattern may reflect inadequate oral hygiene during mixed and permanent dentition, possibly due to fear of gingival bleeding [18,22].

DMFT and dft reflect cumulative caries experience; they do not interchangeably measure current untreated caries or future caries risk. During follow-up, mean DMFT decreased minimally (3.64 to 3.57; *p* = 0.161), which is not expected for a cumulative index. The records did not allow us to verify the cause; variability in the scoring of borderline lesions is one possible explanation. DMFT was therefore interpreted as unchanged and as reflecting baseline caries burden, not an effect of the preventive programme. The decrease in dft (4.38 to 3.47) may reflect the exfoliation of affected primary teeth, which were no longer scored, and does not indicate an effect on caries susceptibility.

Salivary flow rate and buffering capacity influence dental caries, but evidence in patients with haemophilia is limited and suggests no significant differences from controls [16]. Consistent with previous evidence, no significant between-group differences in salivary parameters were observed in the present study. However, salivary flow rate increased significantly within the haemophilia group over six months. Further large-scale studies are needed to clarify the clinical significance of these findings.

*S. mutans* is primarily implicated in the initiation of dental caries, whereas *lactobacilli* contribute to lesion progression [24]. Salivary quantification of these microorganisms is used as a caries-risk indicator. However, dental caries is a multifactorial, biofilm-mediated disease associated with ecological changes in the oral microbial community rather than with individual species alone. Although salivary *S. mutans* and *lactobacilli* counts remain useful indicators in conventional caries risk assessment, they do not provide a comprehensive characterization of oral microbial dysbiosis, and the present microbiological findings should be interpreted accordingly. Clinical and microbiological parameters have also been assessed together in studies of periodontal therapy and domiciliary oral hygiene protocols in other clinical settings; these studies provide supporting background rather than direct evidence for children with haemophilia [25,26,27].

Studies evaluating salivary bacterial levels in patients with haemophilia are limited and report inconsistent findings [16,28]. In the present study, *S. mutans* levels were significantly higher in the haemophilia group at baseline, whereas *lactobacilli* levels did not differ significantly between the groups. In the haemophilia group, both *S. mutans* and *lactobacilli* levels decreased significantly at six months, and *lactobacilli* levels were lower than those of the controls’ baseline values. Because the bacterial levels were assessed semi-quantitatively and the control group was not followed, these changes should be interpreted cautiously. To our knowledge, longitudinal data on salivary S. mutans and *lactobacilli* levels in children with haemophilia are scarce, and the present six-month observations add to this limited evidence. Previous studies on the relationship between disease severity and oral health parameters have reported inconsistent results [18,22]. In the present study, no significant differences among severity subgroups were detected at baseline. At six months, salivary flow rate differed among the subgroups (overall *p* = 0.015). Because the severity groups were very small (31, 9 and 3 participants at baseline; 24, 8 and 2 at follow-up), these analyses were exploratory and provide very limited information, particularly for the mild subgroup. This study did not establish a clear association between disease severity and oral health, and the uncertainty in the smaller subgroups is substantial.

## 5. Limitations

This study has several limitations that should be considered when interpreting the findings. First, the control group was examined only once and was not followed longitudinally; the six-month values of the haemophilia group were therefore compared with the controls’ baseline measurements, which serve as a cross-sectional reference only. Consequently, the improvements observed after follow-up cannot be attributed to the preventive programme alone, as regression to the mean, natural variation, and repeated contact with the clinical team may also have contributed. The preventive programme itself consisted of several components (oral hygiene education, fluoride toothpaste advice, and fluoride varnish application), and the contribution of each component could not be separated. In addition, the haemophilia and control participants were recruited from different settings (a haematology clinic and a dental faculty), which may be associated with differences in dental attendance, parental involvement, and access to care. Adherence to the recommendations was not formally assessed.

Second, approximately 21% of the haemophilia group (9 of 43 participants) was lost to follow-up (reluctance to attend the hospital during the COVID-19 pandemic, *n* = 4; relocation, *n* = 1; socioeconomic reasons, *n* = 1; and dental treatment related to the start of orthodontic treatment, *n* = 3) which may have introduced attrition bias; the baseline characteristics of participants who completed the follow-up and those who did not could not be compared.

Third, the severity subgroups were small and unequally distributed (31, 9, and 3 participants at baseline, and 24, 8, and 2 at follow-up), so these analyses were exploratory and underpowered, and no firm conclusions can be drawn, particularly for mild haemophilia.

Fourth, all participants were male, which limits generalisability to females, and the participants covered a wide age range (4–17 years), with a wider age distribution in the haemophilia group (SD 4.38 vs. 2.34 years) and different stages of dentition, which affected the number of children eligible for DMFT and dft scoring; changes in primary dentition scores may also be influenced by the exfoliation of primary teeth. In addition, age- and dentition-adjusted analyses were not performed in view of the limited sample size.

Fifth, DMFT is a cumulative index of caries experience and is not expected to change substantially within six months; the six-month interval was chosen to avoid delaying the dental treatment needs of children with haemophilia. Accordingly, DMFT findings were interpreted as reflecting baseline caries burden rather than an effect of the preventive programme. The small decreases in DMFT and dft observed at follow-up are not expected for cumulative indices; they most likely reflect variability in scoring between examinations (DMFT) and exfoliation of primary teeth (dft), and the decayed, missing, and filled components were not analysed separately.

Sixth, bacterial levels were assessed using categorical (semi-quantitative) classifications rather than exact counts. In addition, many outcomes were compared without adjustment for multiplicity, so some nominally significant findings, particularly those with *p* values close to 0.05, may have arisen by chance and should be confirmed in larger studies.

Seventh, information on haemophilia treatment regimen (prophylaxis or on demand), factor products and inhibitor status was not available. Because bleeding contributes to the Gingival Index, the observed GI difference may reflect plaque-related inflammation, haemostatic differences, or both.

Eighth, age, dentition stage, dietary habits, fluoride exposure, socioeconomic characteristics and access to dental care could not be adjusted for in the analyses, as no multivariable analysis was performed. Because the participants were aged 4–17 years, differences in dentition stage and cumulative exposure to cariogenic factors may have influenced the comparisons between the haemophilia and control groups.

Finally, haemophilia is a rare condition, and the overall sample size was necessarily limited. Larger, controlled longitudinal studies are needed to confirm these preliminary observations.

## 6. Conclusions

Children with haemophilia showed poorer oral hygiene, higher gingival inflammation, and greater caries experience than healthy controls at baseline. After six months, oral hygiene parameters improved significantly in the haemophilia group and no longer differed significantly from the controls’ baseline values, although the follow-up had no control group. GI and DMFT remained higher in the haemophilia group.

*S. mutans* levels were initially higher in the haemophilia group and decreased significantly during follow-up. *Lactobacilli* levels also decreased significantly and were lower than the controls’ baseline values at six months, whereas salivary flow rate and buffering capacity showed no significant between-group differences. Analyses according to disease severity were exploratory, and because the subgroups were small and unequally distributed, no clear association between severity and oral health status could be demonstrated. Controlled longitudinal studies are needed to confirm these observations.

## Figures and Tables

**Table 1:** Comparison of quantitative data obtained from the haemophilia group at baseline and six-month with the control group. Data are presented as mean ± SD. Independent Samples Test has been performed.

Parameters	Study Group (Mean ± SD)		Study Group (Mean ± SD)		Control Group (Mean ± SD)		Comparison of Study and Control Group (*p* Value)	
	*n*	Baseline Values	*n*	6th Month Values	*n*	Baseline Values	Baseline Values	6th Month Values
**Simplified OHI**	43	1.65 ± 0.73	34	0.95 ± 0.51	40	0.79 ± 0.76	0.0001 *	0.314
**PI**	43	1.48 ± 0.54	34	0.9 ± 0.45	40	0.76 ± 0.72	0.0001 *	0.311
**GI**	43	1.46 ± 0.41	34	1.11 ± 0.36	40	0.21 ± 0.26	0.0001 *	0.0001 *
**Calculus index**	43	0.16 ± 0.31	34	0.04 ± 0.15	40	0.002 ± 0.01	0.001 *	0.108
**DMFT**	32	3.46 ± 3.57	26	3.57 ± 3.82	36	1.52 ± 1.79	0.008 *	0.007 *
**dft**	27	4.14 ± 3.15	21	3.47 ± 2.89	28	3.85 ± 2.70	0.715	0.638
**Salivary flow rate**	43	0.65 ± 0.19	34	0.73 ± 0.24	40	0.67 ± 0.38	0.869	0.396

Controls were examined once; the six-month columns compare the haemophilia group’s six-month values with the controls’ baseline values. * Statistically significant (*p* < 0.05).

**Table 2:** Comparison of baseline and six-month salivary buffering capacity, *S. mutans*, and *lactobacilli* levels between the haemophilia and control groups (Kruskal-Wallis Test has been performed).

Parameters		Study Group Baseline Values (*n*, %)	Study Group 6th Month Values (*n,* %)	Control Group Values (*n*, %)	Comparison of Study and Control Group (*p* Value)	
					Baseline Values	6th Month Value
**Salivary buffering capacity**	**Low**	4, 9.3%	0, 0%	2, 5%	0.398	0.355
	**Moderate**	9, 20.9%	5, 14.7%	7, 17.5%		
	**High**	30, 69.8%	29, 85.3%	31, 77.5%		
	**Total**	43 100%	34 100%	40 100%		
***S. mutans***	**Absent** **(0)**	1, 2.3%	3, 8.8%	5, 12.5%	0.047 *	0.913
	**Low** < 1 × 10^5^ CFU/ml	9, 20.9%	11, 32.4%	14, 35.0%		
	**Moderate** = 1 × 10^5^ CFU/ml	23, 53.5%	18, 52.9%	13, 32.5%		
	**High** > 1 × 10^5^ CFU/mL	10, 23.3%	2, 5.9%	8, 20%		
	**Total**	43 100%	34 100%	40 100%		
***Lactobacilli***	**Absent** **(0)**	8, 18.6%	18, 52.9%	9, 22.5%	0.204	<0.001 *
	**Low** < 1 × 10^5^ CFU/mL	20, 46.5%	14, 41.2%	10, 25%		
	**Moderate** = 1 × 10^5^ CFU/mL	13, 30.2%	2, 5.9%	14, 35%		
	**High** > 1 × 10^5^ CFU/mL	2, 4.7%	0, 0.0%	7, 17.5%		
	**Total**	43 100%	34 100%	40 100%		

Controls were examined once; the six-month column compares the haemophilia group’s six-month values with the controls’ baseline values. Bacterial levels were assessed semi-quantitatively with the CRT Bacteria test (CFU/mL). Kruskal–Wallis test. * Statistically significant (*p* < 0.05).

**Table 3:** Comparison of baseline and six-month quantitative data obtained from the haemophilia group (paired-samples *t*-test). * Statistically significant (*p* < 0.05).

Parameters	*n*	Baseline Values (Mean ± SD)	Six-Month Values (Mean ± SD)	Comparison of Baseline and Six-Month Values (*p* Values)
Simplified OHI	34	1.56 ± 0.62	0.95 ± 0.51	0.0001 *
PI	34	1.44 ± 0.5	0.9 ± 0.45	0.0001 *
GI	34	1.43 ± 0.4	1.11 ± 0.36	0.0001 *
Calculus index	34	0.12 ± 0.19	0.04 ± 0.15	0.022 *
DMFT	26	3.64 ± 3.82	3.57 ± 3.82	0.161
dft	21	4.38 ± 3.12	3.47 ± 2.89	0.087
Salivary flow rate	34	0.66 ± 0.19	0.73 ± 0.24	0.002 *

**Table 4:** Comparison of baseline and six-month qualitative data obtained from the haemophilia group. (Wilcoxon Signed Ranks Test has been performed).

Parameters		Baseline Values (*n*, %)	6-Month Values (*n*, %)	Comparison of Baseline and 6th Month Values
**Salivary buffering capacity**	**Low**	3, 8.8%	0, 0%	0.046 *
	**Moderate**	7, 20.6%	5, 14.7%	
	**High**	24, 70.6%	29, 85.3%	
	**Total**	34 100%	34 100%	
***S. mutans***	**Absent** **(0)**	1, 2.9%	3, 8.8%	0.012 *
	**Low** < 1 × 10^5^ CFU/mL	6, 17.6%	11, 32.4%	
	**Moderate** = 1 × 10^5^ CFU/mL	19, 55.9%	18, 52.9%	
	**High** > 1 × 10^5^ CFU/mL	8, 23.5%	2, 5.9%	
	**Total**	34 100%	34 100%	
***Lactobacilli***	**Absent** **(0)**	5, 14.7%	18, 52.9%	0.001 *
	**Low** < 1 × 10^5^ CFU/mL	16, 47.1%	14, 41.2%	
	**Moderate** = 1 × 10^5^ CFU/mL	11, 32.4%	2, 5.9%	
	**High** > 1 × 10^5^ CFU/mL	2, 5.9%	0, 0.0%	
	**Total**	34 100%	34 100%	

Wilcoxon signed-rank test (paired comparison of baseline and six-month categories). Bacterial levels were assessed semi-quantitatively with the CRT Bacteria test (CFU/mL). * Statistically significant (*p* < 0.05).

**Table 5:** Subgroup analysis of quantitative parameters according to haemophilia severity in the haemophilia group at baseline and six months (One-way ANOVA and the Tukey HSD test). * Statistically significant (*p* < 0.05).

Parameters	Severity of Haemophilia	Baseline *n*	Baseline Values (Mean ± SD)	6-Month *n*	6-Month Values (Mean ± SD)	Intragroup Comparisons According to Severity of Haemophilia Baseline *p*-Value	Intragroup Comparisons According to Severity of Haemophilia 6th Month *p*-Value
Simplified OHI	Severe	31	1.69 ± 0.77	24	0.86 ± 0.58	*p* = 0.554	*p* = 0.134
	Moderate	9	1.66 ± 0.59	8	1.16 ± 0.12		
	Mild	3	1.21 ± 0.76	2	1.16 ± 0.23		
PI	Severe	31	1.51 ± 0.55	24	0.81 ± 0.50	*p* = 0.506	*p* = 0.137
	Moderate	9	1.53 ± 0.44	8	1.16 ± 0.12		
	Mild	3	1.10 ± 0.69	2	1.00 ± 0.00		
GI	Severe	31	1.44 ± 0.40	24	1.06 ± 0.41	*p* = 0.930	*p* = 0.247
	Moderate	9	1.55 ± 0.38	8	1.20 ± 0.11		
	Mild	3	1.33 ± 0.76	2	1.33 ± 0.24		
Calculus Index	Severe	31	0.18 ± 0.35	24	0.04 ± 0.17	*p* = 0.838	*p* = 0.103
	Moderate	9	0.12 ± 0.22	8	0.00 ± 0.00		
	Mild	3	0.11 ± 0.19	2	0.16 ± 0.23		
DMFT	Severe	22	3.50 ± 3.11	16	3.62 ± 3.46	*p* = 0.163	*p* = 0.366
	Moderate	8	4.25 ± 4.83	8	4.25 ± 4.83		
	Mild	2	0.00 ± 0.00	2	0.50 ± 0.70		
dft	Severe	19	3.89 ± 3.24	16	3.31 ± 2.82	*p* = 0.324	*p* = 0.702
	Moderate	6	5.50 ± 2.81	4	3.75 ± 3.86		
	Mild	2	2.50 ± 3.53	1	5.00 ± 0.00		
Salivary flow Rate	Severe	31	0.65 ± 0.19	24	0.67 ± 0.24	*p* = 0.082	*p* = 0.015 *
	Moderate	9	0.73 ± 0.17	8	0.95 ± 0.09		
	Mild	3	0.46 ± 0.11	2	0.55 ± 0.21		

**Table 6:** Subgroup analysis of qualitative parameters according to severity of haemophilia in the haemophilia group at baseline and six-month (Kruskal–Wallis test).

Parameters	Severity of Haemophilia	Baseline *n*	Baseline Mean Rank	6th Month *n*	6-Month Mean Rank	Intragroup Comparisons According to Severity of Haemophilia Baseline *p*-Value	Intragroup Comparisons According to Severity of Haemophilia 6th Month *p*-Value
**Salivary buffering capacity**	Severe	31	21.58	24	17.17	*p* = 0.513	*p* = 0.804
	Moderate	9	21.28	8	17.88		
	Mild	3	28.50	2	20.00		
***S. mutans***	Severe	31	20.92	24	15.79	*p* = 0.547	*p* = 0.148
	Moderate	9	25.67	8	22.94		
	Mild	3	22.17	2	16.25		
***Lactobacilli***	Severe	31	21.60	24	18.17	*p* = 0.270	*p* = 0.760
	Moderate	9	26.11	8	15.50		
	Mild	3	13.83	2	17.50		

## Data Availability

The data supporting the findings of this study are available from the corresponding author upon reasonable request. The data are not publicly available due to privacy/ethical restrictions.
